# Surge in C-section deliveries during the COVID-19 pandemic: insights from a cross-sectional study in Gujarat, India

**DOI:** 10.1136/bmjph-2024-001733

**Published:** 2025-10-17

**Authors:** Farjana Memon, Mahalaqua Nazli Khatib, Deepak Saxena, Komal Shah, Anish Sinha, Ami V Mehta, Claire Heffernan

**Affiliations:** 1 Indian Institute of Public Health, Gandhinagar, Gujarat, India; 2 Jawaharlal Nehru Medical College, Datta Meghe Institute of Higher Education and Research, Maharashtra, Wardha, India; 3 Department of Obstetrics & Gynecology, NHL Municipal Medical College, Ahmedabad, Gujarat, India; 4 Department of Population Health, London School of Hygiene & Tropical Medicine Centre of Global Change and Health, London, UK

**Keywords:** COVID-19, Community Health, Cross-Sectional Studies

## Abstract

**Background:**

The COVID-19 pandemic wreaked havoc and devastated economic, social and healthcare systems worldwide, particularly in resource-constrained countries. Maternal and child health outcomes deteriorated amidst the pandemic due to the pandemic’s multifaceted effects, including the viral infection itself, stringent containment policies and evolving guidelines for antenatal and postnatal care services. While numerous studies have documented the increasing prevalence of C-section deliveries, there remains a dearth of evidence on the factors contributing to this trend during the pandemic. This study aimed to document the trend and contributing factors in the mode of delivery during the COVID-19 pandemic in Gujarat, India.

**Method:**

A cross-sectional survey was carried out on 611 women who delivered during the pandemic (from March 2020 to February 2022); data were collected on the sociodemographic profile, reproductive history, mode of delivery and factors contributing to the overall C-section delivery using the WHO-recommended Robson’s 10-group classification system (TGCS) tool.

**Result:**

Study findings documented a C-section rate of 45.7%, which is significantly higher than the WHO threshold and the latest national average. The primary contributors to the overall C-section rate, as identified by Robson’s TGCS groups, were group 5 (multiparous with previous caesarean section (CS), single, cephalic, ≥37 weeks) followed by group 2 (nulliparous, single cephalic, ≥37 weeks, induced labour or CS before labour) and group 1 (nulliparous, single cephalic, ≥37 weeks, spontaneous labour). Among COVID-19-infected mothers, the C-section rate was even higher, with preterm birth emerging as a third major contributing factor.

**Conclusions:**

These findings underscore the urgent need to address the increasing trend of C-section deliveries, conduct comprehensive analyses targeting these groups, evaluate existing management protocols and conduct further research into indications of C-section and outcomes in the local context. These steps are crucial in our collective efforts to reduce the C-section delivery rate and design tailored strategies.

**WHAT IS ALREADY KNOWN ON THIS TOPIC**

**WHAT THIS STUDY ADDS**

- This study provides valuable insights into the factors contributing to the high rate of C-section deliveries during the pandemic.
- It highlights the necessity for a robust and regular monitoring context-specific tool to track C-section trends.

**HOW THIS STUDY MIGHT AFFECT RESEARCH, PRACTICE OR POLICY**

- This study offers crucial information for policy-makers aiming to reduce unnecessary C-sections.
- By pinpointing the main drivers, healthcare authorities can develop targeted interventions and improve clinical guidelines.

## Introduction

 The COVID-19 pandemic has devastated healthcare systems across the globe, particularly in low-resource countries. Maternal and child health outcomes were among the most severely affected by their inherent vulnerability, infection itself, global efforts to contain the virus, evolving guidelines for antenatal care (ANC) and postnatal care services and restrictions on childbirth delivery options limited to designated health facilities.^1^ Along with other immediate maternal health outcomes, the C-section rate has also increased during the pandemic.^2^

Caesarean section (CS), often abbreviated as C-section, is a surgical procedure that involves fetal delivery through an open abdominal incision (laparotomy) and an incision in the uterus.^3^ The WHO recognises that while C-section can be life-saving for both mothers and babies in medically necessary situations, they should ideally be reserved for complicated pregnancies^4 5^ and should not exceed 10%–15% for any region to maintain optimal perinatal health.^6^ In India, evidence indicates an increase in the percentage of caesarean deliveries over the past decade, surpassing the WHO’s recommended threshold of 15%, which would be a severe public health concern. Surveys such as the National Family Health Survey (NFHS) 4 (2015–2016) and NFHS 5 (2019–2020) have confirmed this increasing trend by reporting 17.2% and 21% C-section rates, respectively.^7 8^ This phenomenon is not unique to only India; other developing countries, such as Bangladesh, China and Sri Lanka, have also seen the same trend of increasing C-section in the past two decades.^5^ Available research during the COVID-19 pandemic has further highlighted the increase in C-section rates. Despite their importance in necessary situations, unnecessary C-sections can pose significant risks for both mothers and babies, leading to increased mortality and morbidity rates, complications and financial burdens on healthcare systems, particularly in low-income and middle-income countries.^9^,^11^ Given this trend, ongoing audits of C-section in healthcare settings are crucial, and effective monitoring tools are essential to understand and address this rise.

The literature has documented the three most commonly adopted classifications or monitoring tools based on primary clinical indications, the degree of urgency and the Robson classification. Among these, Robson’s 10-group classification, also known as the 10-group classification system (TGCS), was optimal for monitoring C-section.^12 13^ This is a WHO-recommended global standard for assessing and comparing C-section rates across healthcare facilities, allowing for mutually exclusive and comprehensive categorisation based on six essential obstetric characteristics.^14^ Therefore, this study aims to document the prevalent mode of delivery and comprehend the factors contributing to the overall mode of delivery (C-section) using such classification frameworks in two districts of Gujarat, India.

## Materials and methods

### Study type

A cross-sectional survey was implemented in the purposively selected study setting. Women who delivered during the COVID-19 pandemic (March 2020–February 2022) in the selected study setting and who consented to participate were recruited for the study.

### Study settings

This study was conducted in the districts of Ahmedabad and Sabarkantha in Gujarat, India. Ahmedabad was characterised as an urban area, and Sabarkantha, designated as a rural district, was chosen to comprehensively understand urban dynamics and rural contexts.

### Sample size and sampling framework

The study included 614 women who delivered between March 2020 and February 2022 across public, private and COVID-19-designated health facilities in the selected study area. Eligible participants were initially identified through facility records based on their delivery period and geographical location and classified as index cases (women who delivered during the pandemic). Due to challenges in direct access to all eligible participants and the lack of a central repository, snowball sampling was adopted to expand the sample size through participant referrals. Using OpenEpi, V.3, the required sample size was 512, considering a 21.1% prevalence rate, 95% confidence level, 1 370 300 live births in 2020–2021 based on a birth rate of 19.3 per 1000 population and design effect of 2. To adjust for a 20% expected non-response rate, the target was set at 614. The actual on-field non-response rate was 0.5%, resulting in 611 participants for analysis, ensuring statistical rigour and minimising bias.

Eligibility criteria:

- Women aged 18–49 years.
- Pregnant during the COVID-19 pandemic in the selected study settings.
- No history of major chronic illnesses such as cancer or immunodeficiency syndromes.
- Willing to provide informed consent to participate.

### Data collection and analysis

The data were collected via personal interviews using a prestructured, pilot-tested tool. This mainly focused on the sociodemographic profile, delivery type, delivery outcome and Robson’s TGCS six obstetric variables comprising parity, the onset of labour, gestation week, fetus presentation, previous CS and the number of gestations. Subsequently, they were segregated into ten distinct groups based on these criteria alongside each group’s contribution to the overall CS rate. The details have been provided in the multiple-choice questionnaire.

SPSS V.20.0 was used to perform the analysis. Descriptive analysis was performed to present the sociodemographic profile and mode of delivery. Inferential analysis was performed to determine the contributing factors among the overall and stratified groups based on COVID-19 infection status during pregnancy.

### Patient and public involvement

This study did not involve the public or patients directly in designing and conducting the research because it was not operationally feasible.

## Result

As shown in table 1, the mean age of the enrolled participants in this study was 28.5±4.4 years. The total CS rate was 45.7% during the COVID-19 pandemic, which is much higher than the WHO recommended threshold and prepandemic period. Again, it was significantly higher in the COVID-19-positive group (62.8%) than in the COVID-19-negative group (37.4%). As usual, the overall C-section rate was higher in the private health facility; however, among the COVID-19-positive group, the CS rate in the public health facility was higher than in the COVID-19-negative group.

**Table 1:** Basic sociodemographic details of mothers recruited during the COVID-19 pandemic

Characteristics	n=611	(%)
Mean age	28.5±4.4	
District		
Ahmedabad (Urban)	450	73.6
Sabarkantha (Rural)	161	26.4
Religion		
Hindu	546	89.4
Muslim	65	10.6
Level of education		
Illiterate	46	7.5
Can read and write	15	2.5
Up to higher secondary	410	67.1
Graduation and above	139	22.7
Ration card		
Above poverty line	357	58.4
Below poverty line	131	21.4
None	122	19.9
Type of delivery		
C-section	279	45.7
Vaginal	332	54.3

Table 2 was documented based on verbal responses for C-section reasoning. As described in Table 2, the COVID-19-positive group opted for more elective C-section deliveries in comparison to the COVID-19-negative group. Both qualitative findings (verbal response) and TGCS group classification documented higher C-section rates among the women with a history of previous C-section; however, the TGC group stratified it according to parity and gave a clearer picture.

**Table 2:** Reasons for C-section delivery

Reasons for CS	COVID-19-positive	COVID-19-negative	P value
Electives	28 (23.0)	16 (10.5)	0.01
Previous CS with/without medical indication	36 (29.5)	33 (21.7)	
Fetal distress/complication	26 (21.3)	47 (30.9)	
Maternal complication/morbidity	30 (24.6)	47 (30.9)	
Twins+with/without mother or fetal complication	2 (1.6)	9 (5.9)	

CS, caesarean section.

Table 3 represents the trend of C-section delivery and contributing factors associated with overall C-section deliveries using Robson’s TGCS classification during the pandemic. A detailed classification of Robson’s TGCS classification is provided in the online supplemental table 1. The findings of this study revealed that the greatest contributing factor to overall CS was group 5 (multiparous women with a history of previous CS, presenting with a single cephalic fetus at ≥37 weeks of gestation), followed by group 2 (nulliparous women with a single cephalic fetus at ≥37 weeks of gestation, with induced labour or CS before labour) and group 1 (nulliparous women classified under group 1 with a single cephalic fetus at ≥37 weeks of gestation, experiencing spontaneous labour). However, there was a disparity of contributory factors, as well as overall trends, between women who tested positive for COVID-19 and those who tested negative, as shown in the online supplemental tables 2,3. The analysis of online supplemental tables 2,3 revealed that while the top two contributory factors showed consistent trends across both groups and the total population, the third highest factor showed remarkable variation among COVID-19-positive women. The third most significant contributory factor among COVID-19-positive women was identified as group 10, characterised by preterm birth irrespective of parity, history of a single cephalic fetus and gestational age less than 37 weeks, including previous CSs. This factor also incorporates findings and observations related to preterm birth data, which were high in the COVID-19-positive group and within the field observation.

**Table 3:** Trend of C-section delivery and contributory factor to overall C-section delivery during the COVID-19 pandemic

Robson’s group	No. of CS over the total number of women in each group	Relative size of group	CS rate in each group	Contribution made by each group to overall CS rate
1	33/113	18.5 (113/611)	29.2	5.4
2	50/59	9.7 (59/611)	84.7	8.2
3	14/182	29.8 (182/611)	7.7	2.3
4	14/16	2.6 (16/611)	87.5	2.3
5	86/97	15.9 (97/611)	88.7	14.1
6	19/19	3.1 (19/611)	100	3.1
7	11/13	2.1 (13/611)	84.6	1.8
8	11/18	2.9 (18/611)	61.1	1.8
9	6/7	1.1 (7/611)	85.7	1.0
10	27/97	12.9 (79/611)	27.8	4.4

CS, caesarean section.

## Discussion

CS) rates have steadily increased worldwide over the past three decades, even before the COVID-19 pandemic. Since 1990, both developed and developing nations have reported a significant increasing trend in the prevalence of C-section deliveries. The rates of caesarean deliveries have more than doubled in India, from 8% of deliveries in 2005 to 17% in 2016. Recent data from 154 countries covering 94.5% of global live births shows that 21.1% of women worldwide had caesarean births with a wide range of variations, from 5% in sub-Saharan Africa to 42.8% in Latin America and the Caribbean.^15^,^17^ Surprisingly, the highest average annual rate of increase (6.4%) is documented in the Asian regions.^18^ With the onset of the COVID-19 pandemic, this rising trend intensified across almost all countries, highlighting a consistent rise in C-section rates during this period.^19^,^21^ Our research further corroborates this, revealing a substantial surge in C-section delivery amidst COVID-19, reaching 45.7%. This increase can be attributed to multiple factors, including disruptions in maternal healthcare services, changes in clinical decision-making and heightened concerns regarding maternal-fetal complications associated with COVID-19.

Notably, this surge was particularly significant among women with a history of COVID-19 infection during pregnancy, where the C-section rate surged to 62.8%, compared with 37.4% in COVID-negative mothers. Similar observations have been documented in studies conducted at rural tertiary care centres in Wardha, Maharashtra, India and rural tertiary care centres in Ghana, which had overall CS rates of 43.4% and 46.9%, respectively.^22^ In contrast, this percentage is slightly higher than that reported in other studies conducted during the third wave of the COVID-19 pandemic at a tertiary teaching hospital in the west coast of Gujarat (28.4%), which showed a decreasing trend in CS in the last 4 years (in 2021: 33.65%; in 2020: 33.28%, in 2019: 33.04%),^23^ and in a study conducted during the same time period in Kurdistan Province, Iran. Additionally, the trend was observed to be unchanged in China and decreased in Iceland.^24^,^26^

The key contributors to the overall C-section rate were determined according to Robson’s classification. We found that the TGCS group 5 emerged as the primary contributing factor, followed by groups 2 and 1. Interestingly, SARS-CoV-2 infection during pregnancy did not alter the distribution of contributing factors within group 5, which consistently remained the highest and most prevalent in both of the groups, that is, those who had and had not received SARS-CoV-2 infection during pregnancy. For group 5, this finding is not an isolation similar to the echo which has been documented in prepandemic and during pandemic situations.^2127^,^31^ On the other hand, research carried out during the third wave of the pandemic in the same state revealed that group 1 had the most contributory factors, followed by group 5.^23^ Further research at the initial stages of the pandemic at Nishtar Medical University Hospital in Multan, Pakistan, highlighted Group 10 as the primary contributor, followed by group 5.^12^ Similarly, a study conducted at a tertiary teaching hospital, Addis Ababa, Ethiopia, documented group 10 as the highest contributor, followed by groups 2 and 5.^32^ Additionally, a study conducted at a Spanish tertiary hospital, which examined both the prepandemic and pandemic periods, identified group 2 as the most significant contributory factor, followed by group 5.^33^ The presented evidence shows that group 5, that is, multiparity with a history of previous C-section, single cephalic presentation and pregnancy at least/more than 37 weeks, remained among the top three contributing factors regardless of the pandemic situation and presence of SARS-CoV-2 infection during pregnancy. Consequently, interventions focusing on this criterion could lower the C-section rate.

The trend in contributory factors to the overall C-section rate remained consistent for the top two factors among women with and without a history of COVID-19 infection. However, the third highest factor differed between the groups. Among COVID-19-infected mothers, preterm births (TGCS group 10, that is, all preterm single cephalic, < 37 weeks including previous CS) emerged as the third highest contributing factor, highlighting the strong link between high preterm birth rates and increased C-sections. This variance could be attributed to the impact of SARS-CoV-2 infection during pregnancy. Based on previous evidence of increased preterm birth risk among pregnant women infected with COVID-19, it is reasonable to speculate that mothers who were affected by COVID-19 infection during the third trimester may have been prompted to opt for early delivery to ensure the safety of both mothers and baby, resulting in higher incidences of preterm births and CSs in this group. Our on-site observations corroborate this hypothesis, as we noticed that most women who tested positive for COVID-19 in the third trimester, particularly those with high RT‒PCR scores, were often advised to proceed with delivery, regardless of whether they had reached the gestational age of 37 weeks.

### Conclusions and recommendations

Our research revealed that the rate of CS significantly increased during the pandemic, with RTGS groups 5, 2 and 1 emerging as primary contributing factors to all CS. To address this, policy-makers should prioritise strengthening clinical guidelines to ensure adherence to evidence-based indications for CS, particularly for high-contributing groups. Routine monitoring and audits using Robson’s classification or any other context-specific tool should be mandated across healthcare facilities to track trends and assess the necessity of procedures. Healthcare providers should receive continuous training on best practices for labour management to reduce unnecessary surgical interventions. Additionally, public health campaigns must focus on disseminating accurate maternal health information through widely accepted and authentic platforms, ensuring accessibility to even remote communities. Lastly, pandemic preparedness strategies should include specific protocols for maintaining high-quality ANC and intrapartum care while preventing an unwarranted surge in C-sections due to misinformation or fear. Implementing these measures will enhance maternal health outcomes and ensure that CS procedures are performed based on medical necessity rather than non-clinical factors.

## Limitations

This study enrolled women delivered during the pandemic; however, comparing the trends separately for each of the waves as well as prepandemic and postpandemic trends in terms of CS would strengthen the evidence. Although Robson’s 10-group classification is widely accepted for monitoring CS rates, it could better reflect the current pandemic situation and enhance its utility by incorporating COVID-19 infection status as an independent category. This addition could provide a robust understanding of CS trends amidst the pandemic, thereby enriching the applicability of the classification system in contemporary research and clinical settings.

## Supplementary material

10.1136/bmjph-2024-001733 online supplemental table 1

## Data Availability

Data are available on reasonable request.
