# Supplementary material for: Surge in C-section deliveries during the COVID-19 pandemic: insights from a cross-sectional study in Gujarat, India
Source: BMJ Public Health. 2025 Oct 17;3(2):e001733. doi: 10.1136/bmjph-2024-001733 (PMC12542720; doi:10.1136/bmjph-2024-001733)
Supplement: online supplemental table 1 [file bmjph-3-2-s001.docx]

| **Class. no** | **Classification details** |
| --- | --- |
| 1 | Nulliparous, single cephalic, ≥ 37 weeks, spontaneous labor |
| 2 | Nulliparous, single cephalic, ≥ 37 weeks, induced labour or CS before labour |
| 3 | Multiparous without previous CS, single, cephalic, ≥ 37 weeks, spontaneous labour |
| 4 | Multiparous without previous CS, single, cephalic, ≥ 37 weeks, induced labour or CS before labour |
| 5 | Multiparous with previous CS, single, cephalic, ≥ 37 weeks |
| 6 | All nulliparous breeches |
| 7 | All multiparous breeches (including previous CS) |
| 8 | All multiple pregnancies (including previous CS) |
| 9 | All transverse or oblique lies (including previous CS) |
| 10 | All preterm single cephalic, < 37 weeks (including previous |

**Table: 1 Robson’s Classification Criteria**

**Table: 2 Trend of C-section delivery among the women who had a history of Covid Infection during Pregnancy**

| **Robson’**  **s group** | **Total no of CS/Total women in each group** | **Relative size of group** | **CS rate in each group** | **Contribution made by each group to overall CS rate** |
| --- | --- | --- | --- | --- |
| 1 | 14/26 | 13.1(26/199) | 53.8 | 7.0 |
| 2 | 29/31 | 14.6 (29/199) | 93.5 | 14.6 |
| 3 | 5/41 | 20.6 (41/199) | 12.2 | 2.5 |
| 4 | 5/7 | 3.5 (7/199) | 71.4 | 2.5 |
| 5 | 38/40 | 20.1 (40/199) | 95.0 | 19.1 |
| 6 | 10/10 | 5.0 (10/199) | 100.0 | 5.0 |
| 7 | 1/1 | 0.5 (1/199) | 100.0 | 0.5 |
| 8 | 2/8 | 4.0 (8/199) | 25.0 | 1.0 |
| 9 | 1/1 | 0.5 (1/199) | 100.0 | 0.5 |
| 10 | 15/29 | 14.6 (29/199) | 51.7 | 7.5 |

**Table 3: Trend of C-section delivery among the women who had a not history of Covid Infection during Pregnancy**

| **Robson’s**  **group** | Number of CS/Total no of women in each group | Relative size of group | CS Rate in Percentage | Contribution of each group to  overall CS rate |
| --- | --- | --- | --- | --- |
| 1 | 19/87 | 21.1 (87/412) | 22 | 4.6 |
| 2 | 21/28 | 6.8 (28/412) | 75 | 5.1 |
| 3 | 9/141 | 34.2 (141/412) | 6 | 2.2 |
| 4 | 9/9 | 2.2 (9/412) | 100 | 2.2 |
| 5 | 48/57 | 13.8 (57/412) | 84 | 11.7 |
| 6 | 9/9 | 2.2 (9/412) | 100 | 2.2 |
| 7 | 10/12 | 2.9 (12/412) | 83 | 2.4 |
| 8 | 9/10 | 2.4 (10/412) | 90 | 2.2 |
| 9 | 5/6 | 1.5 (6/412) | 83 | 1.2 |
| 10 | 12/50 | 12.1 (50/412) | 24 | 2.9 |
